# Stress Levels Among Primary Health Care Workers in Almaty, Kazakhstan: A Cross-Sectional Study

**DOI:** 10.3390/ijerph23030403

**Published:** 2026-03-23

**Authors:** Ainur B. Qumar, Assylkhan Kuttybayev, Mukhtar Kulimbet, Anuarbek Ashikbayev, Akmaral Abikulova, Dimash Davletov

**Affiliations:** 1 Kh. Dosmukhamedov School of Public Health, Asfendiyarov Kazakh National Medical University, 050000 Almaty, Kazakhstan; a.kumar@kaznmu.kz (A.B.Q.); kuttybaev.a@kaznmu.kz (A.K.); 2 Research Management Department, Research Institute of Cardiology and Internal Diseases, 050000 Almaty, Kazakhstan; mkulimbe@kaznmu.kz; 3 School of Dentistry, Asfendiyarov Kazakh National Medical University, 050000 Almaty, Kazakhstan; anuarbek24@icloud.com; 4 Atchabarov Scientific-Research Institute of Fundamental and Applied Medicine, Asfendiyarov Kazakh National Medical University, 050000 Almaty, Kazakhstan; davletov.d@kaznmu.kz

**Keywords:** perceived stress, alcohol, general practitioners, nurses, primary health care, physical activity, smoking

## Abstract

**Highlights:**

**Public health relevance—How does this work relate to a public health issue?**

- Occupational stress among primary health care (PHC) workers is a growing public health concern that affects workforce stability, quality of care, and patient safety.
- Evidence on stress and coping behaviors among PHC workers in Central Asia remains limited, despite ongoing health system reforms.

**Public health significance—Why is this work of significance to public health?**

- This study provides citywide evidence of a high prevalence of perceived stress among PHC workers in Almaty, with general practitioners (GPs) disproportionately affected.
- The findings identify professional role, age, and alcohol abstinence as key factors associated with stress, highlighting vulnerable subgroups within the PHC workforce.

**Public health implications—What are the key implications or messages for practitioners, policy makers, and/or researchers in public health?**

- Organizational interventions aimed at reducing role-related and administrative burden, particularly for GPs, should be prioritized in PHC settings.
- Public health strategies addressing maladaptive coping behaviors, including alcohol use, are needed to support workforce well-being and health system sustainability.

**Abstract:**

Ongoing health system reforms in Kazakhstan have transformed the working environment of primary health care (PHC) staff and may increase workload and psychosocial stress. This study aimed to assess perceived stress among PHC workers in Almaty and its associations with socio-demographic characteristics and health-related behaviors. A cross-sectional survey was conducted in October–November 2023 across all 36 state-funded PHC facilities in Almaty. General practitioners (GPs) and family nurses employed in these facilities were invited to participate. In total, 1484 respondents completed a standardized questionnaire in Kazakh or Russian administered electronically via Google Forms. Perceived stress was assessed using PSS-10, physical activity using IPAQ-SF, alcohol consumption using AUDIT-C, and tobacco use through items aligned with STEPS/GATS. Statistical analyses were performed using SAS. Associations between variables were evaluated using χ^2^ and Fisher’s exact tests, and multivariable logistic regression models were applied. Statistical significance was set at *p* < 0.05. Higher stress levels were more common among GPs than nurses (OR = 2.58; *p* < 0.0001) and less common in younger workers (18–29 vs. 50–59: OR = 0.504; *p* = 0.017) and alcohol abstainers (OR = 0.587; *p* = 0.0004). Kazakh ethnicity showed a borderline protective association (OR = 0.472; *p* = 0.057), while physical activity was not a significant predictor. Perceived stress is highly prevalent in Almaty PHC and disproportionately affects GPs; younger age and alcohol abstinence are protective. The findings support prioritizing organizational measures to reduce role-related burden and maladaptive coping behaviors.

## 1. Introduction

The global healthcare landscape is currently navigating a period of unprecedented volatility, characterized by the convergence of shifting epidemiological burdens, rapid technological disruption, and the lingering systemic aftershocks of the COVID-19 pandemic [1,2,3]. Within this complex ecosystem, Primary Health Care (PHC) serves as the foundational bedrock of effective health service delivery, a principle enshrined in the 1978 Alma-Ata Declaration [4,5]. However, the workforce tasked with sustaining this critical infrastructure faces mounting pressures that threaten both individual well-being and system-wide sustainability [3,6,7]. In low- and middle-income countries (LMICs), and particularly within the post-Soviet states of Central Asia, these challenges are magnified by a unique constellation of historical, economic, and structural factors [8,9,10,11]. The transition from the centralized, specialist-dominated Semashko model of the Soviet era to a modern, family-medicine-based system has necessitated a fundamental rewriting of professional roles, often without a commensurate adjustment in resources or psychosocial support [10,11]. This structural dissonance has created a fertile ground for perceived stress, professional burnout, and maladaptive coping behaviors among health care workers, phenomena that are increasingly recognized not merely as personal grievances but as critical determinants of patient safety, quality of care, and workforce retention [3,6,7].

The Republic of Kazakhstan represents a paradigmatic case study of a health system in flux. Since gaining independence in 1991, the nation has embarked on a series of ambitious reforms to modernize its healthcare infrastructure and achieve Universal Health Coverage (UHC) [8,9]. These reforms have included the introduction of the Unified National Health System, the implementation of Mandatory Social Health Insurance, and a rigorous push toward digitalization through platforms such as “DamuMed” and the “Electronic Health Passport” [10,11,12,13,14,15]. Although these initiatives aim to improve efficiency and access, they have fundamentally altered the daily labor of General Practitioners (GPs) and nurses [7,12,13,14]. The shift has concentrated immense administrative and clinical responsibility at the PHC level, transforming polyclinics into high-pressure environments where providers must navigate complex insurance protocols, manage chronic disease registries, and master evolving digital tools, often while serving large, empaneled populations [7,12,13,14,15]. The reform fatigue generated by these ongoing systemic overhauls and financial inequality is a palpable yet under-quantified force within the sector, potentially exacerbating the risk of professional exhaustion and attrition [3,10,11].

Despite ongoing reforms and documented structural pressures within the Kazakhstani health system [8,9,10,11], empirical evidence specifically addressing perceived stress among PHC workers remains limited. Available national data have primarily focused on professional burnout rather than perceived stress as a distinct psychological construct [16]. Moreover, previous studies in Kazakhstan have not examined perceived stress using standardized instruments such as the PSS-10 within a large urban PHC setting, nor have they systematically explored its association with behavioral risk factors such as alcohol consumption, smoking, and physical activity [16,17].

Behavioral and lifestyle factors such as physical activity, smoking, and alcohol consumption have been shown to be associated with perceived stress and psychological well-being in working populations. Prior research in general working samples has demonstrated that higher perceived stress is correlated with harmful health-risk behaviors, including smoking and alcohol use, as well as lower levels of physical activity [18,19]. For example, higher stress levels were associated with increased prevalence of smoking and other health-risk behaviors in a large cross-sectional study of adult workers, suggesting that stress may influence both coping strategies and lifestyle patterns [20]. Similarly, physical exercise has been identified as a protective factor that may mitigate the relationship between stress and unhealthy behaviors among healthcare professionals [19]. Including these behavioral variables therefore strengthens the theoretical framework of the study by capturing modifiable factors that may co-occur with stress and impact overall health outcomes in PHC workers.

While perceived stress among PHC workers has been widely studied in Western and Middle Eastern settings [21,22], comparable data from Central Asia and post-Soviet health systems remain sparse. Global evidence indicates high levels of burnout and psychological distress among healthcare professionals, with physicians experiencing particularly elevated prevalence rates across diverse healthcare systems [22]. Similarly, nursing staff are vulnerable to burnout due to prolonged exposure to high workload, emotional labor, and limited autonomy, highlighting profession-specific stressors that interact with systemic pressures [23]. While burnout reflects chronic occupational stress and emotional exhaustion, perceived stress is a broader psychological construct that captures the individual’s appraisal of life demands relative to coping resources [24,25,26].

The PSS-10 measures general perceived stress, reflecting both professional and personal life demands, rather than exclusively workplace-related stressors [25]. This distinction justifies the use of the PSS-10 in the present study, as it encompasses both occupational and non-occupational stressors among PHC workers in Almaty.

The present study aims to assess perceived stress among primary health care workers in Almaty and to examine its associations with socio-demographic and behavioral factors.

This study provides the first large-scale citywide assessment of perceived stress among PHC professionals in Almaty using the validated PSS-10 instrument. Second, it examines stress within the context of health system transformation and digitalization, offering insight into role-specific vulnerability among GPs and nurses. Also, by integrating behavioral risk factors, the study extends beyond descriptive prevalence estimates and identifies modifiable correlates of stress relevant to health policy and workforce sustainability.

## 2. Materials and Methods

### 2.1. Study Design and Setting

This cross-sectional study was conducted in Almaty, Kazakhstan, between October and November 2023. It represents a secondary analysis within a broader research project investigating lifestyle behaviors and related health factors among primary health care professionals.

The survey was implemented in all 36 state-funded primary health care facilities in the city and targeted GPs and family nurses, given their central role in preventive counseling and health promotion.

### 2.2. Study Population and Recruitment

All eligible GPs and family nurses employed at the participating facilities were invited to participate. According to official staffing records of the Almaty City Public Health Department, 973 general practitioners and 2065 nurses were employed across the centers at the time of the study.

A total of 1484 individuals completed the questionnaire (response rate 48.8%). The nursing group primarily included family nurses working directly with GPs; procedural and specialized nurses were excluded to maintain consistency with the study’s focus on frontline primary care providers.

Recruitment was conducted in coordination with the Almaty City Public Health Department. Following institutional approval, individual facilities were contacted and study visits were scheduled in agreement with clinic management to minimize disruption to routine activities. Eligible staff received information about the study prior to participation.

### 2.3. Survey Administration

The questionnaire was self-administered electronically using Google Forms (Google LLC, Mountain View, CA, USA). When necessary, assistance was provided to ensure accessibility, while all responses were recorded in a uniform electronic format.

The survey was anonymous, and no identifying personal information was collected. Participation was voluntary, and written informed consent was obtained prior to completion. Respondents were free to skip any questions.

The instrument was available in Kazakh and Russian, and participants selected their preferred language. The average completion time was approximately 45 min.

The questionnaire included structured items on socio-demographic characteristics, physical activity, and alcohol consumption. The study followed established recommendations for online survey design [27] and reporting standards for survey research [28,29]. Prior to implementation, the instrument was pilot-tested among health professionals and refined accordingly.

Detailed information regarding the overall survey framework has been described previously [16].

### 2.4. Variables and Measures

Sociodemographic variables comprised age (18–29, 30–39, 40–49, 50–59 years), gender (male, female), ethnicity (Kazakh, Russian, other), marital status (widowed, never married, divorced, married including civil marriage, or in partnership), work experience (0–10 years and ≥11 years), and professional role (general practitioner or nurse).

The Perceived Stress Scale (PSS), developed by Cohen and colleagues, is designed precisely to assess this global stress appraisal rather than exposure to specific events and has been widely applied in occupational and healthcare settings [24,29,30]. Conceptually, PSS items reflect two interrelated dimensions: perceived helplessness (negatively worded items, e.g., lack of control, overload) and perceived self-efficacy (positively worded items, e.g., confidence in handling problems), which enables a more nuanced interpretation of stress profiles beyond a single total score [25,31]. The PSS-10 showed good internal consistency in the present sample (Cronbach’s α = 0.86; 95% CI: 0.85–0.87). Based on the total PSS-10 score, stress levels were categorized as low (0–13 points), moderate (14–26 points), and high (27–40 points). We merged moderate and high scores to present a group with stress, while the low stress score was defined as no stress group.

Physical activity was assessed using the International Physical Activity Questionnaire Short Form (IPAQ-SF), with classification into low, moderate, or high levels based on MET scores, following official scoring guidelines [32]. Low PA—no or minimal activity not meeting higher thresholds; Moderate—≥3 days of vigorous activity (≥20 min/day) or ≥5 days of moderate activity/walking (≥30 min/day) or any equivalent combination achieving ≥600 MET-min/week; High—≥3 days of vigorous activity totaling ≥1500 MET-min/week or 7 days of any combination of activities achieving ≥3000 MET-min/week.

Alcohol consumption among primary healthcare (PHC) workers was assessed using the AUDIT-C questionnaire [33] based on its brevity and suitability for large-scale surveys. This questionnaire evaluates the frequency of alcohol consumption, the number of alcoholic drinks consumed on a typical day, and the frequency of consuming more than six standard drinks per day. The AUDIT-C includes three questions from the original AUDIT questionnaire, each scored from 0 to 4, resulting in a possible total score of 0 to 12. Hazardous drinking [34], which refers to a pattern of alcohol use that increases the risk of negative consequences, was defined based on AUDIT-C criteria as scores above 5 for men and above 4 for women [35]. Standard drinking was defined as 10 g of pure alcohol.

Smoking status was assessed as overall tobacco use without differentiating between conventional cigarettes, e-cigarettes, hookah, or other products through self-reported questions included in the study questionnaire. Participants were asked whether they currently smoked (yes/no), the average number of cigarettes smoked per day, and their smoking duration. Only three smoking-related questions were used to reduce respondent burden and ensure feasibility in a large-scale survey, while still capturing the core indicators sufficient for epidemiological analyses and comparable with WHO STEPS and GATS data [36,37].

The survey was conducted in Russian and Kazakh. The Russian version relied on previously validated translations, while the Kazakh version was translated and pilot-tested with 10 healthcare professionals to ensure clarity.

### 2.5. Data Analysis

Statistical analysis was performed using SAS OnDemand for Academics (Release 3.81, Cary, NC, USA). Descriptive statistics were used to summarize categorical variables (frequency and percentage). Associations between variables were assessed using Chi-square and Fisher’s exact tests. Multivariable logistic regression was applied to examine predictors of hazardous alcohol use and insufficient physical activity. A *p*-value < 0.05 was considered statistically significant.

## 3. Results

Sample Characteristics and Stress Prevalence A total of 1484 healthcare professionals participated in the study. Based on their PSS-10 scores, participants were divided into two groups: “No stress” (low stress) and “Stress” (medium and high stress).

Response patterns across PSS-10 items were consistent with the overall classification: participants in the stress group more frequently endorsed response categories indicating higher perceived stress (e.g., “sometimes”, “quite often”, or “very often”), particularly for items related to perceived lack of control and task overload. In contrast, participants in the no-stress group more commonly selected response options reflecting confidence and perceived control.

### 3.1. Socio-Demographic and Professional Characteristics

Univariate analysis revealed significant differences in stress prevalence according to age (*p* = 0.0003), ethnicity (*p* = 0.0006), and marital status (*p* = 0.0064). Among the professional groups, GPs demonstrated a significantly higher frequency of stress compared to nurses (*p* < 0.0001). A professional tenure of more than 11 years was also associated with increased stress levels (*p* = 0.0216). Sex (*p* = 0.118) and type of organization (*p* = 0.063) were not statistically significantly associated with stress status (Table 1).

### 3.2. Lifestyle and Alcohol Consumption (AUDIT)

Smoking status (*p* = 0.0004) and physical activity levels (IPAQ, *p* = 0.0599) are summarized in Table 2.

Alcohol consumption patterns, measured by the AUDIT scale, were closely linked to stress levels (*p* < 0.0001). Individuals who do not consume alcohol were significantly less likely to belong to the “Stress” group compared to those who consume alcohol. Specifically, there was a growing tendency to report stress among workers who reported higher frequency, quantity and heavy drinking episodes (Table 3).

### 3.3. Multivariable Analysis of Stress Predictors

A multivariable logistic regression model was constructed to identify independent predictors of high stress levels (Table 4). Professional role remained the strongest independent predictor: GPs had 2.58 times higher odds of experiencing high stress compared to nurses (OR = 2.58; *p* < 0.0001). Being in the 18–29 age group reduced the odds of developing stress by 50% compared to the 50–59 age group (OR = 0.50; *p* = 0.017). Additionally, alcohol abstinence was independently associated with a 41% reduction in the odds of high stress (OR = 0.59; *p* = 0.0004).

## 4. Discussion

The findings indicate that perceived stress among primary health care workers in Almaty is shaped by occupational hierarchy, generational differences, and behavioral patterns. Physicians, particularly older practitioners, appear to carry a disproportionate psychological burden. These results suggest that stress in this setting is embedded not only in individual characteristics but also in structural and organizational factors within the health system.

GPs in Kazakhstan face a “double burden” leading to high stress due to their central role in care coordination, combined with high patient volumes and mandatory digital reporting (e.g., DamuMed), which creates technological stress and administrative hurdles [7,14]. Unlike nurses, GPs bear disproportionate administrative and decision-making liability, indicating that task-shifting has not fully alleviated physician cognitive load [6,16]. Older healthcare workers are the most stressed; educated under the Soviet system, they struggle to adapt to rapid shifts such as MSHI and digital platforms, while younger, digital-native staff are more adaptable [8,9,10,11,12,13]. Stress is strongly correlated with maladaptive coping, particularly hazardous behaviors like alcohol and smoking, especially among GPs. This suggests a hidden curriculum in which systemic pressures drive chemical coping rather than organizational support. The gender-based differences in these behaviors between the predominantly female nursing workforce and the mixed GP workforce may also reflect sociocultural norms.

Global healthcare worker stress is high (30–50%+), with nurses often reporting higher burnout in Western settings due to low autonomy [3,7,13]. However, the Almaty study’s finding of significantly higher stress in doctors aligns with systems undergoing rapid corporatization, such as the US Veterans Health Administration, suggesting physicians bear the brunt of administrative burdens in this Kazakhstani context [38]. Our study contradicts common Western findings that show younger doctors have the highest burnout [39]. This suggests that in post-Soviet economies, older workers struggle more to adapt to new paradigms than younger workers do when entering the professional world. Reduced student debt may also lower young doctors’ financial stress. This finding, however, conflicts with other data from Kazakhstan showing young PHC workers had higher emotional exhaustion, highlighting the difference between perceived stress and burnout [16]. Regionally, high GP stress resonates with Central Asian trends, indicating that while Kazakhstan may have better infrastructure than some neighbors, digitalization introduces psychological costs without user-centric design [7,38]. Almaty’s observed alcohol consumption aligns with Kazakhstan’s higher regional prevalence, necessitating distinct public health responses.

The acute stress burden on GPs necessitates an accelerated implementation of task-shifting policies. While Kazakhstan has introduced the “advanced practice nurse” model legislatively, the practical implementation remains uneven. The data suggests that the current division of labor is unsustainable. Policy makers must move beyond nominal role expansion to functional empowerment. This involves legal and technical adjustments: granting nurses independent access to write entries in DamuMed, allowing them to authorize repeat prescriptions for chronic patients, and giving them autonomy over specific patient cohorts (e.g., healthy child check-ups). Such measures would directly alleviate the administrative bottleneck that currently traps GPs and distribute the cognitive load more equitably across the team. Similar research among resident doctors has shown high levels of perceived stress linked to adverse work conditions, suggesting that stress-management interventions and improvements in the work environment are strongly recommended [40].

### 4.1. Limitations

Despite offering valuable insights into the characteristics and habits of healthcare professionals, the study has several inherent limitations. Firstly, because the research used a cross-sectional design, establishing a cause-and-effect relationship between occupational roles and health behaviors is not possible; thus, any observed associations are correlational, not causal. Secondly, relying on participants to self-report lifestyle information, such as alcohol and tobacco consumption or level of physical activity, introduces the risk of social desirability bias and imperfect recall, which could lead people to minimize socially frowned-upon behaviors or exaggerate health-conscious actions. Thirdly, this study assessed general perceived stress using the PSS-10, which captures non-specific psychological stress rather than stress exclusively originating from the workplace. Therefore, differences observed between GPs and nurses cannot be definitively attributed to occupational stressors alone. Factors outside the professional setting, such as personal or societal demands, may also contribute to the observed stress levels. Finally, the lack of longitudinal data prevents tracking behavioral changes over time or assessing the effectiveness of interventions. Nevertheless, these constraints notwithstanding, the results establish a strong base for developing focused public health initiatives and identify key areas for subsequent investigation.

### 4.2. Future Research

A priority for future research is establishing a longitudinal cohort study of healthcare workers. Tracking a cohort of new medical and nursing graduates as they enter the PHC system would allow researchers to pinpoint the exact timing and causes of the stress onset. Qualitative inquiries, such as focus groups and ethnographic observations, are needed to understand the burden among PHC workers. Usability studies comparing DamuMed’s interface with the cognitive workflows of older vs. younger physicians could inform software redesigns that directly alleviate worker fatigue.

## 5. Conclusions

In a citywide cross-sectional survey of PHC workers in Almaty, a high burden of perceived stress was observed, with physicians experiencing markedly greater stress than nurses. In multivariable analysis, being a GP was the strongest independent predictor of stress (OR = 2.58), whereas younger age (18–29 years) and alcohol abstinence were associated with lower odds of stress (OR = 0.50 and OR = 0.59, respectively). These findings suggest that stress in Almaty PHC is shaped primarily by role-related workload and system-level pressures, and support targeted organizational interventions, especially for GPs, alongside prevention of maladaptive coping behaviors.

## Figures and Tables

**Table 1:** Socio-demographic and professional characteristics of participants by stress status.

Category	No Stress *n* (%)	Stress *n* (%)	Total (*n*)	*p*-Value (χ^2^)
**Sex**				
Female	342 (95.26)	1058 (94.04)	1400	0.3837
Male	17 (4.74)	67 (5.96)	84	
**Age groups**				
18–29	153 (42.62)	350 (31.11)	503	0.0003
30–39	71 (19.78)	274 (24.36)	345	
40–49	65 (18.11)	200 (17.78)	265	
50–59	70 (19.50)	301 (26.76)	371	
**Ethnicity**				
Others	24 (6.69)	141 (12.53)	165	0.0006
Kazakh	327 (91.09)	930 (82.67)	1257	
Russian	8 (2.23)	54 (4.80)	62	
**Marital status**				
widower (widow)	18 (5.01)	66 (5.88)	84	0.0064
never been married	108 (30.08)	225 (20.04)	333	
divorced	36 (10.03)	129 (11.49)	165	
I am married (including civil)	189 (52.65)	679 (60.46)	868	
I am in partnership with 1 partner	5 (1.39)	17 (1.51)	22	
I am in partnership with more than 1 partner	3 (0.84)	7 (0.62)	10	
**Profession**				
General practitioner (GP)	64 (17.83)	407 (36.18)	471	<0.0001
Nurses	295 (82.17)	718 (63.82)	1013	
**Experience**				
0–10 years	204 (56.82)	561 (49.87)	765	0.1200
11–20 years	60 (16.71)	205 (18.22)	265	
21–30 years	42 (11.70)	154 (13.69)	196	
31–40 years	38 (10.58)	165 (14.67)	203	
More than 40	15 (4.18)	40 (3.56)	55	
**Experience grouped**				
0–10 years	204 (56.82)	561 (49.87)	765	0.0216
>11 years	155 (43.18)	564 (50.13)	719	

**Table 2:** Association of smoking status and physical activity with stress prevalence.

Category	No Stress *n* (%)	Stress *n* (%)	Total (*n*)	*p*-Value (χ^2^)
**Do you go to a fitness club?**				
Yes	58 (16.16)	118 (10.51)	176	0.0040
No	301 (83.84)	1005 (89.49)	1306	
**IPAQ**				
High	42 (11.70)	150 (13.33)	192	0.0599
Low	184 (51.25)	496 (44.09)	680	
Moderate	133 (37.05)	479 (42.58)	612	
**Have you ever used tobacco products, such as cigarettes, cigars, e-cigarettes, hookahs?**				
No	308 (85.79)	867 (77.07)	1175	0.0004
Yes	51 (14.21)	258 (22.93)	309	
**How many cigarettes do you smoke per day?**				
0 per day	327 (91.09)	1009 (89.85)	1336	0.1822
≤10 cigarettes	27 (7.52)	78 (6.95)	105	
>10 per day	5 (1.39)	36 (3.21)	41	

**Table 3:** Alcohol consumption patterns (AUDIT) and their association with stress status.

Category	No Stress *n* (%)	Stress *n* (%)	Total (*n*)	*p*-Value (χ^2^)
**AUDIT**				
Abstainer	258 (71.87)	621 (55.20)	879	<0.0001
Low-risk	85 (23.68)	412 (36.62)	497	
Hazardous/harmful	10 (2.79)	42 (3.73)	52	
Likely dependence	6 (1.67)	50 (4.44)	56	
**Total**	**359 (100)**	**1125 (100)**	**1484**	

**Table 4:** Multivariate logistic regression analysis of independent predictors for high stress.

Effect	OR	95% CI (Lower)	95% CI (Upper)	*p*-Value
IPAQ High vs. Moderate	1.037	0.687	1.566	0.862
IPAQ Low vs. Moderate	0.782	0.598	1.023	0.073
AUDIT: Abstainer vs. Low-risk consumption	0.587	0.437	0.788	0.0004
AUDIT: Hazardous/harmful vs. Low-risk consumption	0.912	0.427	1.948	0.812
AUDIT: Likely alcohol dependence vs. Low-risk consumption	2.148	0.874	5.282	0.096
Q502: Yes vs. No	1.416	0.989	2.029	0.058
Q2: Female vs. Male	1.188	0.644	2.192	0.581
Q3: 18–29 vs. 50–59	0.504	0.287	0.884	0.017
Q3: 30–39 vs. 50–59	0.640	0.405	1.011	0.056
Q3: 40–49 vs. 50–59	0.682	0.456	1.021	0.063
Q4: Other vs. Russian	1.069	0.442	2.584	0.882
Q4: Kazakh vs. Russian	0.472	0.218	1.021	0.057
Q6: GP (Family Physician) vs. Nurse	2.579	1.870	3.556	<0.0001
Q7n: 0–10 years vs. ≥11 years	1.185	0.768	1.827	0.443
Q5: Widowed vs. >1 partner	1.295	0.282	5.936	0.739
Q5: Never married vs. >1 partner	0.867	0.209	3.602	0.844
Q5: Divorced vs. >1 partner	1.094	0.253	4.729	0.904
Q5: Married (incl. civil) vs. >1 partner	1.285	0.312	5.298	0.729
Q5: 1 partner vs. >1 partner	1.172	0.204	6.742	0.859

## Data Availability

The data presented in this study are not publicly available due to ethical and privacy restrictions but are available from the corresponding author upon reasonable request.

## Abbreviations

The following abbreviations are used in this manuscript:

PHC	Primary Health Care
PSS	Perceived Stress Scale
IPAQ-SF	International Physical Activity Questionnaire–Short Form
AUDIT-C	Alcohol Use Disorders Identification Test–Consumption
STEPS	WHO STEPwise Approach to Surveillance
GATS	Global Adult Tobacco Survey
